# As old as the hills: Pliocene palaeogeographical processes influence patterns of genetic structure in the widespread, common shrub *Banksia sessilis*

**DOI:** 10.1002/ece3.7127

**Published:** 2020-12-28

**Authors:** Heidi Maria Nistelberger, Sarah‐Louise Tapper, David J. Coates, Shelley L. McArthur, Margaret Byrne

**Affiliations:** ^1^ Department of Biodiversity, Conservation and Attractions Biodiversity and Conservation Science Bentley WA Australia

**Keywords:** cpDNA, Darling Scarp, evolution, microsatellites, phylogeography

## Abstract

The impact of Quaternary glaciation on the development of phylogeographic structure in plant species is well documented. In unglaciated landscapes, phylogeographic patterns tend to reflect processes relating to persistence and stochasticity, yet other factors, associated with the palaeogeographical history of the landscape, including geomorphological events, can also have a significant influence. The unglaciated landscape of south‐western Western Australia is an ideal location to observe these ancient drivers of lineage diversification, with tectonic activity associated with the Darling Fault in the late Pliocene attributed to patterns of deep phylogeographic divergence in a widespread tree from this region. Interestingly, other species within this region have not shown this pattern and this palaeogeographical boundary therefore presents an opportunity to examine age and historical distribution of plant species endemic to this region. In this study, we assess patterns of genetic diversity and structure across 28 populations of the widespread shrub *Banksia sessilis* using three cpDNA markers and nine nuclear microsatellite markers. Sixteen cpDNA haplotypes were identified, comprising two major chloroplast DNA lineages that are estimated to have diverged in the Pliocene, approximately 3.3 million years ago. This timing coincides with major geomorphological processes in the landscape, including the separation of the Darling Plateau from the adjacent Swan Coastal Plain, as well as eustatic changes on the Swan Coastal Plain that are likely to have resulted in the physical isolation of historical plant lineages. Chloroplast lineages were broadly aligned with populations associated with older lateritic soils of the Darling Plateau and Geraldton sandplains or the younger sandy soils associated with the Swan Coastal Plain and Southern Coastline. This structural pattern of lateritic versus non‐lateritic division was not observed in the nuclear microsatellite data that identified three genetic clades that roughly corresponded to populations in the North, South, and Central portions of the distributions.

## Graphical abstract

Phylogeographic structure in *Banksia sessilis* coincides with the uplift and separation of the Darling Plateau from the Swan Coastal Plain during the Pliocene.

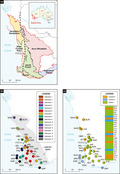

## 1 INTRODUCTION

The development of genetic structure in plant species results from the complex interaction between demographic, climatic, and ecological processes that occur over multiple timescales (Cox & Moore, 2005). In younger, glaciated landscapes characteristic of the northern hemisphere, patterns of genetic diversity in plants have been heavily influenced by the repeated contraction and expansion of populations in response to the glacial/interglacial cycles of the Pleistocene (Hewitt, 2000, 2004; Petit et al., 1997; Tab erlet et al., 1998). These processes resulted in signatures of higher genetic diversity in pockets of the landscape that remained unglaciated and regions of lower genetic diversity where populations expanded following glacial retreat, although patterns of recolonization have varied depending on ecological requirements, such as substrate (Alvarez et al., 2009).

In landscapes that remained unglaciated throughout the Pleistocene, plant species have shown different patterns of genetic diversity and structure that reflect the influence of localized historical persistence. These species often possess remarkably high levels of genetic diversity in comparison to those of glaciated landscapes (Soltis et al., 2006; Sork et al., 2016). This is attributed to long periods of persistence allowing for accrual of genetic variation and limited extinction preserving that variation through time (Cowling & Lombard, 2002; Harrison & Noss, 2017). In addition, many species, including those with widespread distributions, exhibit strong phylogeographic structure. In these instances, well‐defined and highly divergent lineages may reflect processes of historical isolation that are no longer evident in the current distribution, representing localized responses to shifts in suitable habitat over time as well as stochastic processes associated with persistence over long time frames (Byrne et al., 2008, 2019).

In some cases, ancient species may retain signatures of tectonic and orogenic processes that date back to the Oligocene (González‐Martínez et al., 2010; Magri et al., 2007). For example, Magri et al., (2007) identified patterns of geographical genetic structure in cork oak (*Quercus suber*) populations that was consistent with the break‐up of the European‐Iberian continental margin during the Oligocene and Miocene, suggesting the species has persisted on several separate microplates undergoing genetic drift for up to 15 million years. The same pattern was observed in the mitochondrial DNA of the long‐lived tree *Pinus pinaster* (Burban & Petit, 2003). In Taiwan, temporal patterns of phylogeographic structure in *Hygrophila pogonocalyx* coincide with physical isolation caused by the formation of the Central Mountain Range approx. 5 million years ago (Huang et al., 2005). These studies highlight how relictual taxa can harbor signatures of geomorphological events that predate the climatic oscillations of the Quaternary that are so frequently implicated in the generation of plant genetic structure.

The unglaciated South Western Australian Floristic Region (SWAFR) is an ideal location to search for signatures of palaeogeographical processes associated with geomorphological events in plant taxa. Species in this landscape including those that are widespread are often characterized by remarkably high levels of genetic diversity and divergence in the slowly evolving cpDNA genome (Llorens et al., 2017; Nistelberger et al., 2014; Sampson et al., 2018; Wheeler & Byrne, 2006). This reflects their ancient origins and persistence throughout Pleistocene climatic fluctuations, which in the absence of glaciation, involved oscillating periods of warm/wet and cool/dry climatic conditions (Byrne et al., 2008). Strong phylogeographical structuring has even been observed in species that experience high levels of contemporary gene flow (Llorens et al., 2017; Sampson et al., 2018; Wheeler & Byrne, 2006).

**Figure 1:**
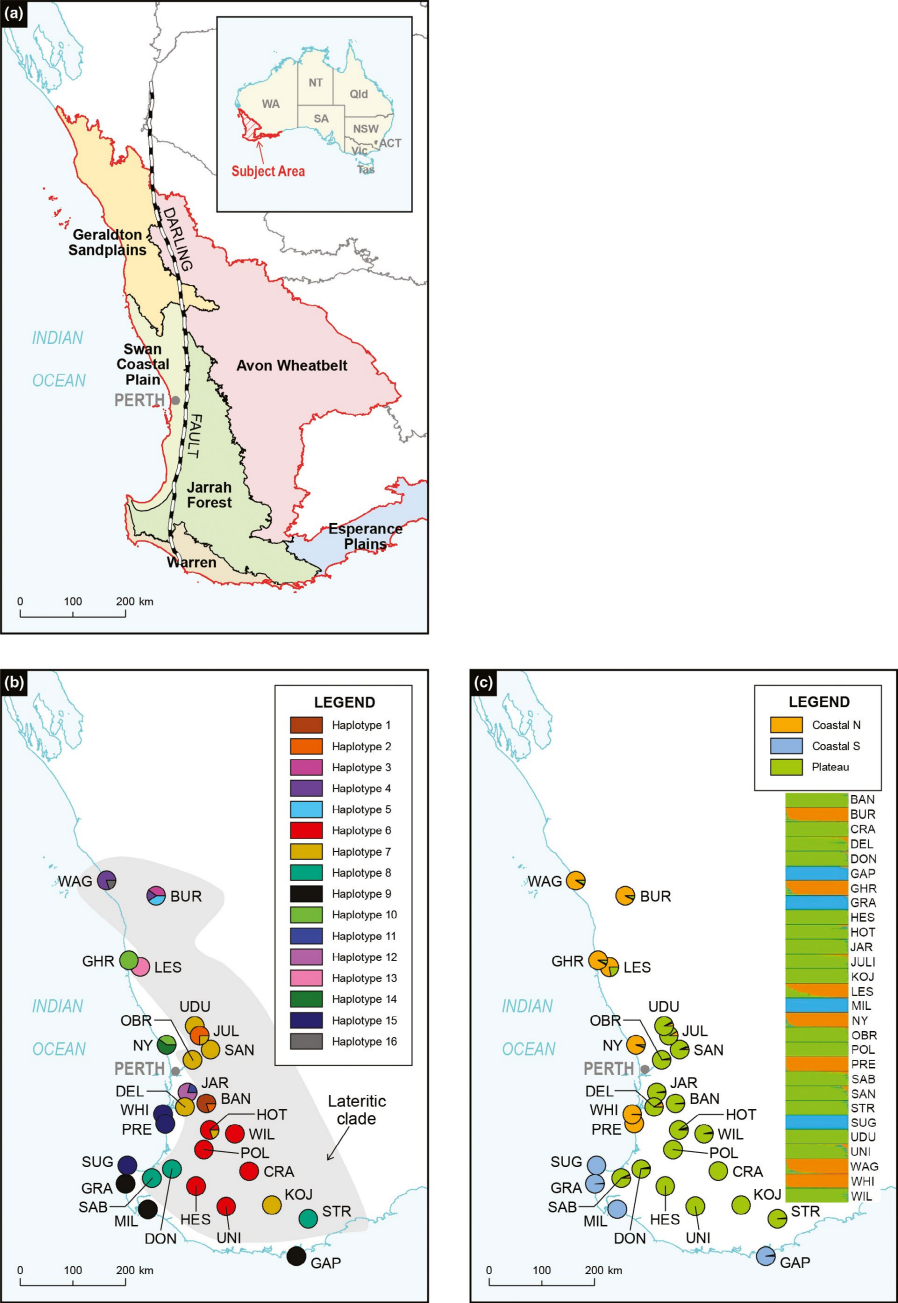
Maps showing; (a) Southern Western Australian IBRA Bioregions; (b) the locations of the 28 *Banksia sessilis* populations sampled and their corresponding chloroplast DNA haplotypes determined via sequencing of *trnS*‐*trnG*, *trnT*‐*psbD,* and *pet B*. Shaded and unshaded regions represent the Lateritic and Non‐lateritic clades identified, respectively; (c) pie charts and bar plot colored according to the assignment of each individual to the three genetic clusters identified using nuclear microsatellite data in STRUCTURE, labeled North Coastal, South Coastal, and Plateau

The most obvious geological feature of the SWAFR is the Darling Fault which occurs in a north–south direction for approximately 1,000 km and is bounded by the Perth Basin to the west that extends to the edge of the continental crust, and the Darling Plateau to the east that encompasses the Archean Yilgarn Block (Beard, 1979; Playford et al., 1976) (Figure 1). Overlaying the Perth Basin is the Swan Coastal Plain (SCP), a strip of eolian and alluvial sands that range in age from late Pliocene in the east to the Holocene in the west (McArthur & Bettenay, 1974; Playford et al., 1976; Seddon, 1972). Faultline activity that commenced in the mid‐Neogene resulted in the gradual uplifting of the Darling Plateau and its separation from the Coastal Plain, and by the Pleistocene an exposed Darling Scarp formed coastal cliffs (Playford et al., 1976). Subsequently, the Perth Basin was uplifted and subject to repeated marine inundation as sea levels rose and fell in association with glacial/interglacial cycles of the Pleistocene (Williams et al., 1993). These sea‐level fluctuations, in addition to the physical separation of the Darling Plateau from the SCP, are anticipated to have impacted the distribution of coastal flora of the SWAFR, with periods of higher sea levels marooning populations on higher elevation islands, and lower sea levels allowing population expansion into regions 30 km westward of the current coastline of the Indian Ocean (Nevill et al., 2014; Pickett & Newsome, 1997; Raymo et al., 2011; Seddon, 1972). The younger soils of the SCP contrast to those of the older, more stable Darling Plateau that overlays the Yilgarn Craton and encompasses the Jarrah Forest, Avon Wheatbelt and Esperance Plains bioregions, and the Geraldton Sandplains bioregion to the north, that underwent massive laterization throughout the Oligocene and Miocene (Bettenay, 1984; Johnstone et al., 1973). Although each biogeographic region is associated with unique vegetation communities, there are widespread species that occur across the different regions.

A study of the widespread tree species *Eucalyptus marginata* identified two phylogeographic lineages that coincided spatially and temporally, with geological activity resulting in the separation of the SCP from the Darling Plateau in the late Neogene (Wheeler & Byrne, 2006). This geomorphological process was hypothesized as the major driver of phylogeographic structure in *E. marginata* (Wheeler & Byrne, 2006). Interestingly, other species from this landscape with similar distributions (i.e., *Corymbia calophylla* (Sampson et al., 2018), *Allocasuarina humilis* (Llorens et al., 2017) and *Calothamnus quadrifidus* (Nistelberger et al., 2014) have not shown these signatures, and instead display younger phylogeographic lineages shaped by Pleistocene forces that occurred after the separation of the SCP and Darling Plateau. Consequently, the study of widespread plant species that cover this palaeogeographical boundary provides an opportunity to explore patterns of age and historical distribution in this landscape.

*Banksia sessilis* (Knight) AR Mast & KR Thiele (formerly *Dryandra sessilis*) is a common and widespread woody shrub endemic to the SWAFR. The species occurs across multiple biogeographic regions including the sandy SCP and southern coastal sands, the lateritic duplex soils of the Darling Plateau, and the lateritic and non‐lateritic sands of the Geraldton Sandplains. Habitat types vary from Kwongan heath in the Geraldton Sandplains to Jarrah forest on the Darling Plateau and Banksia woodland on the SCP. The species straddles the biogeographical boundary created by the Darling Fault and so is an ideal candidate to assess hypotheses of phylogeographic structure resulting from palaeogeographical processes. Intrinsic factors, such as local distribution, mating system and fire response, may also influence genetic diversity. We have no a priori reasons to expect differences in these factors across the distribution, and so predict that the widespread distribution of the species in locally abundant populations, and visitation by mobile pollinators, will lead to little differences in genetic diversity and connectivity across the distribution. Using a combination of cpDNA and nuclear microsatellite markers, we investigated patterns of genetic diversity and structure at historical and contemporary time scales and test hypotheses that: (a) *Banksia sessilis* will show patterns of phylogeographical structure concordant with palaeographical processes associated with the uplift of the Darling Plateau; and (b) the current widespread distribution will reflect high levels of genetic connectivity across the landscape as revealed in contemporary genetic markers.

## 2 MATERIALS AND METHODS

### 2.1 Study species and environment

*Banksia sessilis* is a woody shrub that produces prolific bird‐pollinated flowers and seed that are a food source for parrots and cockatoos (Collins et al., 2008; Lamont et al., 1998). Honeyeaters, wattlebirds, and spinebills are the dominant pollinators throughout the long flowering period that runs from April to October (Collins et al., 2008). *Banksia sessilis* has a widespread distribution across south‐western Australia encompassing variable substrate, elevation, and climate. It occurs on sand and limestone common in the coastal areas, as well as on coastal laterite deposits in the north of the distribution. It is also common along the lateritic Darling Scarp and inland on the Darling Plateau that rises approx. 300 m from the coastal plain. Soils on the Darling Plateau are a mixture of duplex sand over clay and laterite. The species is often dominant where it occurs and responds favorably to disturbance (Rockel et al., 1982). It is killed by fire and regenerates prolifically from seed (Lamont et al., 1998).

Four taxonomic varieties are currently recognized within the species (George, 1996). Two of the four varieties (var. *cordata* and var. *cygnorum*) are coastal and grow in alkaline sands over limestone, while two (var. *sessilis* and var. *flabellifolia*) occur further inland on acid, sandy or duplex soils over laterites. Morphological differences between varieties are limited to minor differences in the shape and color of leaves and presence of hairs on the stem. While plants from many populations sampled could be identified as belonging to one of the four recognized varieties, plants at several sites could not be readily ascribed variety status according to current morphological descriptions. We therefore conducted all analyses at the species level, *Banksia sessilis*.

### 2.2 Sampling, sequencing, and genotyping

Leaf samples were collected from 24 individuals from each of 28 populations sampled. For each population, adult plants were sampled within a 500 m^2^ area, with care being taken to avoid sampling plants that were near‐neighbors and therefore potentially related. Collected leaves were immediately placed on silica gel and later freeze‐dried. DNA was extracted from approximately 50 mg of freeze‐dried material using a modified 2% CTAB method (Doyle & Doyle, 1990), outlined in Nistelberger et al. (2015).

Sequence variation in chloroplast DNA (cpDNA) regions known to detect intraspecific variation in Australian plants (Byrne & Hankinson, 2012) were assessed in a subset of individuals from the extremes of the geographic range. The three most variable regions, *trnS*‐*trnG*, *trnT‐psbD,* and *pet B*, were selected for sequencing on five, randomly selected individuals from each of the 28 populations. Amplification was carried out as in Byrne and Hankinson (2012) and cleaned products were sequenced using the *trnS, trnT*, and *pet B* (sak23F) universal primers by Macrogen (South Korea). Sequences were aligned using BIOEDIT v. 7.2 (Hall, 1999), and all mutations were checked against raw sequence reads. Samples from two closely related species *B. echinata* (A. S. George) and *B. hewardiana* ((Meisn.) A.R. Mast & K. R. Thiele), as well as a specimen of *Macadamia integrifolia* were included as outgroups.

DNA samples from 24 individuals were genotyped using 10 nuclear microsatellite loci developed for the species (Nistelberger, McArthur, et al., 2015). Fragments were amplified according to Nistelberger, McArthur, et al. (2015), and polymerase chain reactions were multiplexed in three sets of four loci using a Qiagen Multiplex PCR Kit in a total volume of 7.5 µl. Fragment analysis was carried out on an Applied Biosystems 3730 DNA Analyzer (Murdoch University), and genotypes were scored manually in Genemapper TM v.5 (Applied Biosystems).

### 2.3 Data analysis—chloroplast data

Chloroplast haplotypes, haplotype diversity, and nucleotide divergence among clades were calculated using DnaSP v5.10 (Librado & Rozas, 2009). For all analyses posthaplotype inference, indels were coded for manually as transitions. Nucleotide diversity (Jukes and Cantor averaged over all loci) and partitioning of genetic variation within and among populations (AMOVA) was determined using ARLEQUIN v3.5 (Excoffier & Lischer, 2010). Tests of neutrality including Tajima's D and Fu's *F*s as well as mismatch analyses testing for signatures of demographic expansion and contraction were also conducted in ARLEQUIN with significance determined using Goodness of fit tests on Harpending's raggedness index and the sum of squared differences. The *R*_2_ statistic, which displays greater power when working with smaller sample sizes (Ramos‐Onsins & Rozas, 2002), was calculated in the R package PEGAS (Paradis, 2010). Phylogenetic relationships were inferred with a Bayesian analysis conducted in BEAST v.1.7.5 (Drummond et al., 2012). A GTR + gamma model was applied following identification as the appropriate model of sequence evolution using the Akaike information criterion in JMODELTEST v. 0.1.1 (Posada, 2008). Four independent runs of 10 million generations were performed, using a strict molecular clock owing to the relatively low level of variability in the cpDNA data and a Yule tree prior (Heled & Drummond, 2012). The tree was dated using the basal divergence between the Banksieae and *Macadamia* at 62 Ma according to He et al. (2011) with *Macadamia integrifolia* (Genbank acc. KF862711.1) used as an outgroup. A normal prior distribution was set around the divergence time with a standard deviation of 3.1 million years. Trees were visualized with FigTree v1.4.4 (http://tree.bio.ed.ac.uk/software/figtree/). The relationships among haplotypes were also investigated with a maximum parsimony analysis (Median‐Joining method) implemented in NETWORK v.4.6.1.3 (Fluxus Technology, Suffolk, UK).

### 2.4 Data Analysis – nuclear data

For the nuclear microsatellite data, tests of genotypic disequilibrium among pairs of loci were conducted in GENEPOP v.4.0.11 (Raymond & Rousset, 1995) and the presence and frequency of null alleles was assessed using a maximum likelihood method in ML‐NULL (Kalinowski & Taper, 2006). Diversity statistics for populations, Nei's unbiased genetic distance (Nei, 1978), and the number of effective migrants (Nm) among population pairs were calculated in GENALEX v.6.5 (Peakall & Smouse, 2012). The fixation index of individuals relative to subpopulations (*F*_IS_) and pairwise population differentiation values (Weir and Cockerham's 1984 *F*_ST_) were determined in GENEPOP with significance determined with 9,999 exact tests. Pairwise population *F*_ST_ values were displayed in R using the “pheatmap” package (Kolde, 2018). We evaluated the performance of four relatedness estimators (Li et al., 1993; Lynch & Ritland, 1999; Queller & Goodnight, 1989; Wang, 2002) in the R package RELATED (Pew et al., 2015). The nonlikelihood moment estimator of Wang (2002) provided the best Pearson's correlation coefficient between observed and expected relatedness values for each estimator (*R* = 0.764) and was used to analyze relatedness within populations. Simulations (*n* = 100) of dyads were performed to determine the distribution of *R* values expected for the data in Parent‐offspring, full sibling, half sibling, and unrelated relationships. The partitioning of genetic diversity among populations was tested with an analysis of molecular variance (AMOVA) implemented in ARLEQUIN v.3.5. One‐way ANOVAs were carried out to determine the effect of genetic cluster identification on mean genetic diversity estimates.

Genetic structure among individuals and populations was visualized using Bayesian clustering analysis implemented in STRUCTURE v.2.3.4 (Pritchard et al., 2000). The STRUCTURE analysis was run for one million generations after a burn‐in of 100,000 generations and utilized an admixture ancestry model with correlated allele frequencies due to the widespread distribution of this species throughout the landscape. Simulations of genetic clusters (K) between 1 and 29 were run, with 10 iterations, and the posterior mean estimates recorded. STRUCTURE HARVESTER (http://taylor0.biology.ucla.edu/structureHarvester/) was used to determine the most likely number of genetic clusters based on the Evanno method (Evanno et al., 2005). To ensure stationarity had been reached, convergence was then assessed using CLUMPP v.1.1.2 (Jakobsson & Rosenberg, 2007). As an alternative to methods that use biological assumptions to estimate genetic distance (Goldstein & Pollock, 1997), the geometric chord distances of Cavalli‐Sforza (*D*_C_) (Cavalli‐Sforza & Edwards, 1967) were also used to reconstruct population relationships as a neighbor joining tree in the program POPULATIONS v.1.2.30 (Langella, 2000). Genetic structure was also visualized with a Principal Coordinates Analysis implemented in the R package ADEGENET (Jombart & Ahmed, 2011). Isolation by distance was investigated using a Mantel test with 9,999 permutations on matrices of log‐geographic distance and population genetic differentiation (*F*_ST_/(1 − *F*_ST_) in GENALEX.

## 3 RESULTS

### 3.1 Chloroplast sequence data

The concatenated, aligned cpDNA sequence data (2,992 bp inc. indels) for *B. sessilis* were comprised of 13 varying length indels, six transitions, 15 transversions, one multistate substitution, and three mononucleotide repeats (that were removed from analysis). This resulted in 16 haplotypes from a total of 138 sequences (one individual failed to amplify in each of the JULI and LES populations). Most populations (*n* = 21) possessed just one haplotype. Seven populations possessed more than one haplotype and six populations possessed population‐specific haplotypes (Table 1, Figure 1). Haplotype frequency ranged from 0.007 (Hap 11, 16) to 0.21 for the common haplotype (Hap 6). Haplotype diversity (HD) was higher in the lateritic clade (0.792 ± 0.03) than the non‐lateritic clade (0.758 ± 0.02), but nucleotide diversity (Pi) was higher in the non‐lateritic clade (0.170 ± 0.09 versus 0.109 ± 0.06) (Table 2).

**Table 1:** Diversity statistics based on nine nuclear microsatellite markers for 28 populations of *Banksia sessilis* with assignment to each microsatellite genetic cluster identified

Pop	Cluster	*N*	Na	Pa	Ne	*H*_O_	*H*_E_	*F*_IS_	RelW	Hap#
BUR	North Coastal	22.4	5.33	4	2.75	0.427	0.516	0.181**	0.02	3,4,5
GHR	North Coastal	22.6	5.56	4	2.74	0.394	0.476	0.155**	0.08*	10
LES	North Coastal	23.2	3.78	1	2.25	0.422	0.433	0.030*	0.22*	13
NY	North Coastal	23.3	6	4	3.17	0.389	0.471	0.183**	0.00*	10,14
PRE	North Coastal	23.6	3.56	1	2.15	0.378	0.434	0.150**	0.24*	15
WAG	North Coastal	23.4	4.78	3	2.68	0.5	0.531	0.074**	0.02*	4,16
WHI	North Coastal	23.7	4	3	2.41	0.367	0.459	0.207**	0.13*	15
mean	North Coastal	23.2 (0.2)	4.72 (0.33)	2.9 (0.5)	2.59 (0.12)	0.411 (0.02)	0.474 (0.01)	0.14 (0.02)	0.10 (0.03)	
GAP	South Coastal	22.8	2.56	1	1.49	0.167	0.266	0.375**	0.38*	9
GRA	South Coastal	23.8	3.22	0	1.93	0.313	0.34	0.102*	0.33*	9
MIL	South Coastal	23.7	2.67	0	1.84	0.32	0.337	0.076	0.43*	9
SUG	South Coastal	22.6	2.89	1	1.7	0.317	0.295	−0.05	0.54*	15
mean	South Coastal	23.2 (0.27)	2.84 (0.13)	0.5 (0.3)	1.74 (0.08)	0.279 (0.03)	0.310 (0.02)	0.13 (0.08)	0.42 (0.04)	
BAN	Plateau	23.7	3.22	0	1.96	0.393	0.398	0.033*	0.25*	1,2
CRA	Plateau	23.7	3.67	0	2.46	0.39	0.403	0.054	0.17*	6
DEL	Plateau	23.3	3.44	0	2.31	0.394	0.481	0.177**	0.10*	7
DON	Plateau	23.6	2.56	0	1.64	0.306	0.32	0.065**	0.46*	8
HES	Plateau	23.4	2.56	1	1.55	0.284	0.278	0	0.54*	6
HOT	Plateau	23.4	3.67	0	1.92	0.329	0.369	0.124	0.22*	6,7
JAR	Plateau	23.8	2.89	0	1.69	0.289	0.317	0.102	0.41*	11,12
JULI	Plateau	23	3.67	0	2.2	0.374	0.387	0.061*	0.18*	2,7
KOJ	Plateau	23.4	3.11	0	2.05	0.39	0.38	−0.01	0.28*	7
OBR	Plateau	24	4.11	0	2.14	0.343	0.393	0.148**	0.20*	7
POL	Plateau	23.2	2.56	0	1.82	0.388	0.375	−0.026	0.37*	6
SAB	Plateau	21.3	2.67	2	1.8	0.4	0.411	0.041*	0.38*	8
SAN	Plateau	23.6	3.44	1	1.83	0.347	0.366	0.075*	0.30*	7
STR	Plateau	22.6	3	0	1.94	0.371	0.335	−0.079	0.30*	8
UDU	Plateau	22.7	3.78	0	2.57	0.477	0.475	0.012	0.15*	7
UNI	Plateau	24	2.33	0	1.72	0.329	0.311	−0.034	0.39*	6
WIL	Plateau	23.8	4	0	2.24	0.408	0.447	0.108*	0.14*	6
mean	Plateau	23.3 (0.16)	3.22 (0.13)	0.2 (0.1)	1.99 (0.07)	0.365 (0.01)	0.379 (0.01)	0.05 (0.02)	0.28 (0.02)	

Note

*N*, mean number of individuals genotyped per locus; Na, number of alleles; Pa, number of private alleles; Ne, number of effective alleles; *H*_O_, observed heterozygosity; *H*_E_, expected heterozygosity; *F*_IS_, inbreeding coefficient; Relatedness estimate (Wang, 2002); Hap#, cpDNA haplotype number. Standard errors in parentheses.

* *p* < .05.

** *p* < .005.

**Table 2:** Diversity statistics, tests of neutrality, and mismatch analyses based on chloroplast data consisting of concatenated *trnS*‐*trnG*, *trnT*‐*psbD,* and *pet B* sequences for *Banksia sessilis*

	Non‐lateritic clade	Lateritic clade	All populations
# Populations	9	19	28
# Haplotypes	6	10	16
Haplotype diversity	0.758 (0.03)	0.792 (0.02)	0.880 (0.01)
Nucleotide diversity	0.170 (0.09)	0.109 (0.06)	0.236 (0.12)
Tajima's D	2.15	1.39	1.33
Fu's *Fs*	7.609	4.009	6.126
*R*_2_	0.152	0.058	0.074
Demographic expansion	SSD (*p* = .00): Hrag (*p* = .00)	SSD (*p* = .02): Hrag (*p* = .01)	SSD (*p* = .11): Hrag (*p* = .00)
Spatial expansion	SSD (*p* = .14): Hrag (*p* = .32)	SSD (*p* = .34): Hrag (*p* = .43	SSD (*p* = .17): Hrag (*p* = .11)

Note

Number of haplotypes including indels; Haplotype Diversity; Nucleotide diversity ‐ Jukes and Cantor averaged over loci, standard deviation in parentheses; Tajima's D; Fu's *Fs*; *R*_2_; Mismatch analyses testing for patterns of demographic and spatial expansion, SSD = sum of squared differences, Hrag = Harpending's raggedness index. Number of haplotypes including indels; Haplotype Diversity; Nucleotide diversity ‐ Jukes and Cantor averaged over loci, standard deviation in parentheses; Tajima's D; Fu's *Fs*; *R*_2_; Mismatch analyses testing for patterns of demographic and spatial expansion, SSD = sum of squared differences, Hrag = Harpending's raggedness index.

**Figure 2:**
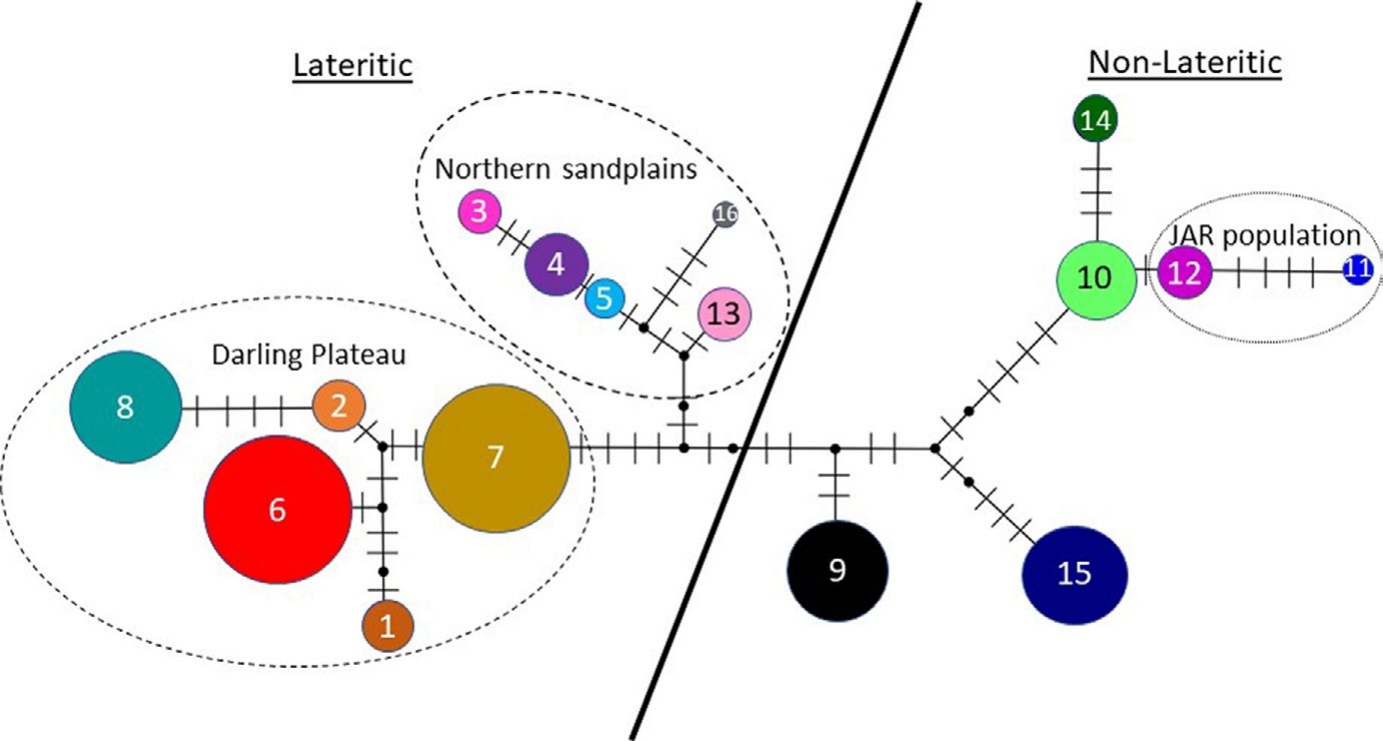
Median‐joining maximum parsimony network of the 16 *Banksia sessilis* cpDNA haplotypes identified (colored according to Figure 1b). Node size is proportional to haplotype frequency and dashes represent mutational changes. Solid black line indicates separation of the two highly supported clades as identified in the phylogenetic analysis. Note haplotypes found in the Jarrahdale (JAR) population showed affiliation with the non‐lateritic clade despite occurring on laterite

**Figure 3:**
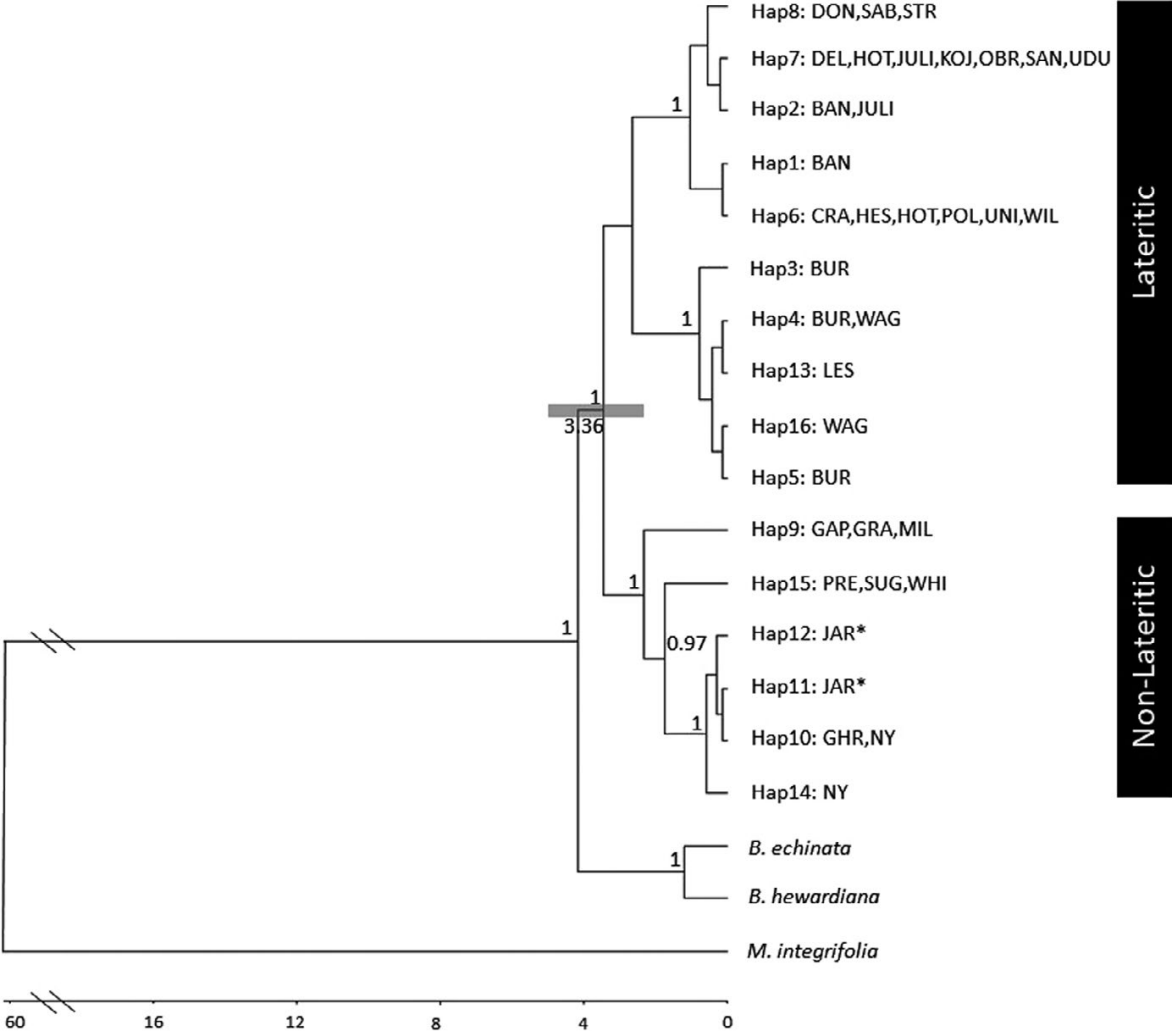
Phylogenetic tree of 16 cpDNA haplotypes of *Banksia sessilis*. Numbers in red indicate posterior probabilities >95%. Lower horizontal bar indicates time in years before present. Asterisk highlights the JAR population haplotypes that cluster with the non‐lateritic clade despite the population occurring on laterite

The BEAST analysis resulted in the identification of two well‐supported clades, broadly corresponding to populations present on lateritic soils associated with the Darling Plateau and Geraldton Sandplains (hereafter lateritic clade) and those found on sand and limestone associated with the geologically younger coastal region of the SCP and Southern Coastline (hereafter non‐lateritic clade). There was one exception to this trend; haplotypes 11 and 12 from the Jarrahdale (JAR) population (Figure 1), located on lateritic soils of the Darling Scarp, clustered with haplotypes present in the non‐lateritic clade (Figure 2), a relationship also supported by the phylogenetic tree (Figure 3). Molecular dating using the basal divergence between the Banksieae tribe and the outgroup *Macadamia* places the divergence of the two clades at 3.3 Ma (2.0–5.0 Ma) during the Pliocene (Figure 3). The network analysis showed high levels of divergence among haplotypes particularly those occurring in the non‐lateritic populations (Figure 2).

Within clades, most haplotypes were restricted to single, or geographically proximate populations, although four were more widespread. Two of these were from the lateritic clade (Hap 6 and Hap 7) and were widespread across the Darling Plateau whereas Haplotype 9 and 15 from the non‐lateritic clade were widespread across the southern coastal sands and SCP respectively. Haplotype diversity was higher in the lateritic clade, but nucleotide diversity was higher in the non‐lateritic clade. (Table 2). AMOVA indicated 58.6% of the genetic variation was found among clades, 38.4% maintained among populations within a clade and only 3% within populations, reflecting the dominant pattern of single haplotypes per population. Nucleotide divergence (Jukes and Cantor) between the two clades was 0.263%.

Estimates of neutrality (Tajimas's D, Fu's *Fs* and *R*_2_) that may also predict population size changes were nonsignificant in both clades, as well as when considering all populations as a group (Table 2). Mismatch analysis showed no evidence for sudden demographic expansion in either clade, but all populations together showed some evidence under the SSD test. In contrast, both clades, and all populations as a group, showed evidence of spatial expansion in both the SSD and Hrag tests (Table 2, Figure S1).

### 3.2 Microsatellite data

One of the loci (Bs45) showed a high frequency of null alleles (36.8%) and was associated with populations where many or all individuals failed to amplify and was therefore removed from the dataset. The remaining nine loci showed no evidence of genotypic disequilibrium following Bonferroni correction. The average number of alleles per population ranged from 2.33 to 6 (Table 1). The number of effective alleles per population was lowest in the GAP population (1.49) and highest in the NY population (3.17) (Table 1). Observed and expected heterozygosity were also lowest in the GAP population (0.167 and 0.266, respectively) and highest in the northern WAG population (0.500 and 0.531, respectively) (Table 1).

**Figure 4:**
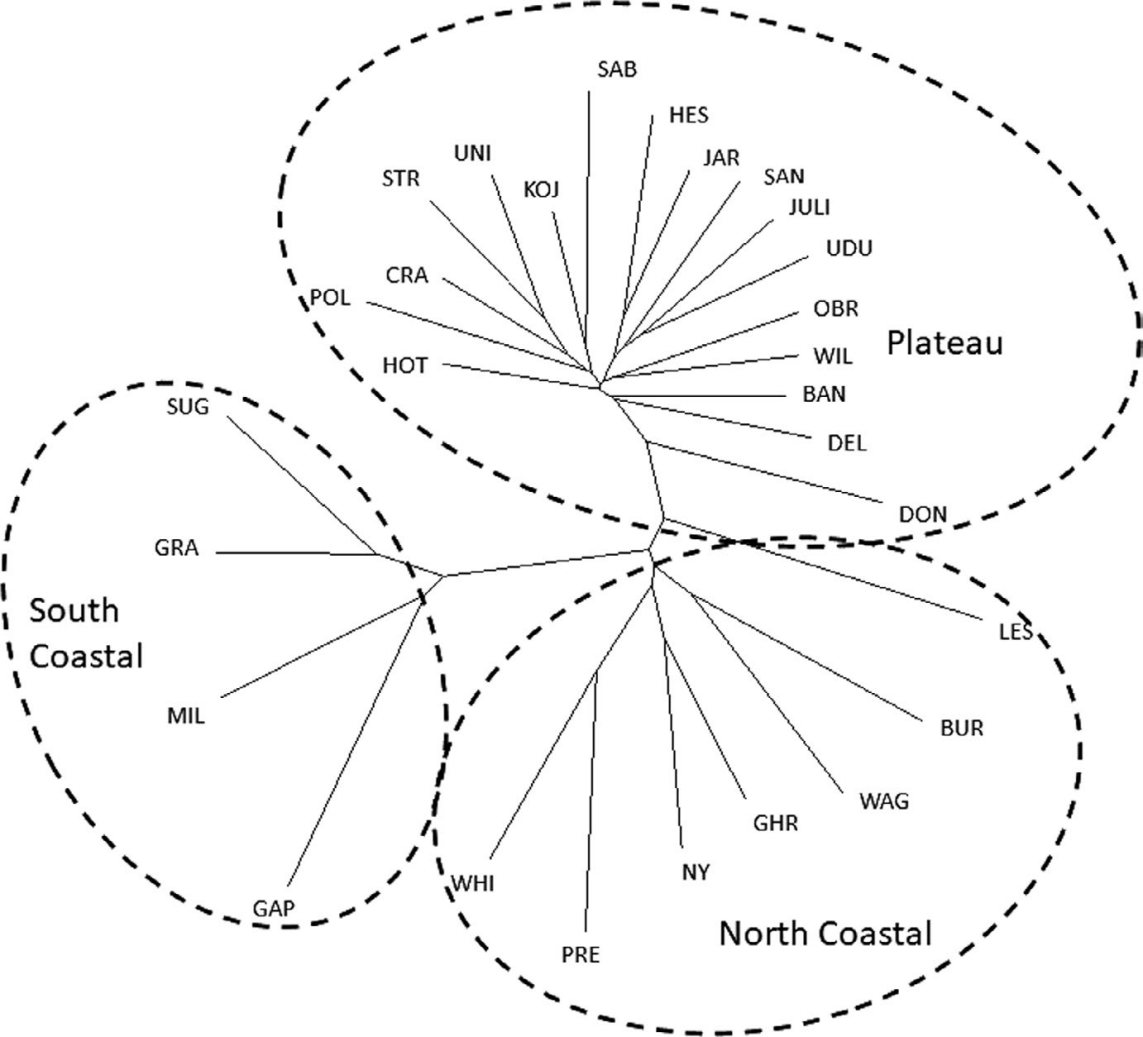
Neighbor joining tree of geometric Cavalli‐Sforza chord distances (*D*_C_) calculated among the 28 populations of *Banksia sessilis* using microsatellite data. Population assignment according to microsatellite data is indicated to enable comparison

**Figure 5:**
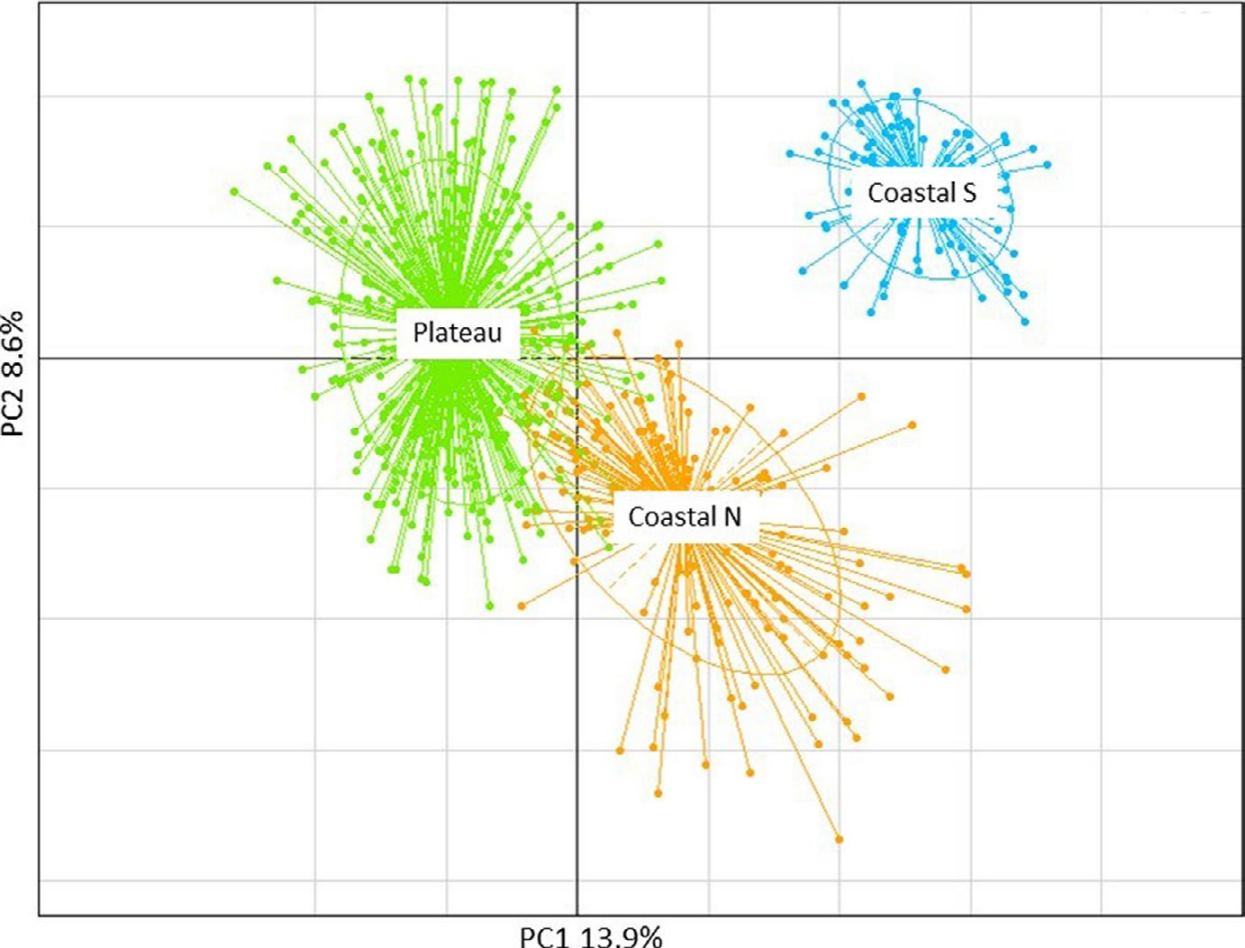
Principal coordinates analysis of *Banksia sessilis* individuals based on data from nine nuclear microsatellites and colored according to the genetic clusters identified in the STRUCTURE analysis (Figure 1c). Axes show the amount of variance explained by each principal component (PC)

STRUCTURE analysis identified three genetic clusters (*K* = 3) that did not reflect the lateritic/non‐lateritic division identified in the cpDNA data. Instead, the three groups broadly corresponded to populations occurring on the lateritic Darling Plateau (hereafter Plateau), those on sands, limestone, and laterite associated with the northern coastline (from PRE northward to WAG) (hereafter North Coastal), and those associated with sands and limestone of the southern coastline (from SUG southward to GAP) (hereafter South Coastal) (Figure 1). Admixture was evident between several populations from the North Coastal and Plateau populations (Figure 1c). The findings for *K* = 2 and *K* = 4 are presented in Appendix S1 for comparison (Table S1, Figure S2).The neighbor joining tree of populations based on Cavalli‐Sforza chord distances showed an identical geographic association of the three genetic clusters with some intergrade between North Coastal and Plateau populations (Figure 4) and principal coordinates analysis confirmed segregation of the three clusters, with some overlap between North Coastal and Plateau populations and complete isolation of South Coastal populations (Figure 5). Analyses of variance indicated genetic diversity was significantly higher in the North Coastal group in estimates of mean number of effective alleles (Ae) (*F*_(2,25)_ = 13.67, *p *= <.005), mean observed heterozygosity (*H*_O_) (*F*_(2,25)_ = 8.22, *p *= <.005), and mean expected heterozygosity (*H*_E_) (*F*_(2,25)_ = 15.32, *p *= <.005). Estimates of *F*_IS_ were often high, with significant estimates observed in 17 of the 28 populations (Table 1). Mean estimates were significantly higher in the North Coastal and South Coastal clades (*F*_(2,25)_ = 3.08, *p *= <.005). All populations except BUR showed significantly higher levels of relatedness than expected based on 500 randomized iterations (Table 1). Relatedness among population individuals was significantly higher in populations from the South Coastal clade (*F*_(2,25)_ = 10.88, *p *= <.005) where the mean estimate indicated the presence of full sibling and parent–offspring relationships among individuals (Table 1. Figure S3).

Populations varied in their degree of genetic differentiation with *F*_ST_ values as low as 0.03 observed between GHR and NY, two North Coastal populations approx. 170 km apart, to a high *F*_ST_ of 0.61 between GAP and HES, populations from the South Coastal and Plateau clades, respectively, located approx. 210 km apart (Table S2). Within clusters, mean genetic differentiation was highest among South Coastal populations (*F*_ST_ = 0.32 ± 0.03; Nei's *D*_ST_ = 0.278 ± 0.03) in comparison with North Coastal populations (*F*_ST_ = 0.17 ± 0.02; Nei's *D*_ST_ = 0.232 ± 0.02) and Plateau populations (*F*_ST_ = 0.17 ± 0.01; Nei's *D*_ST_ = 0.136 ± 0.01). Across all populations, the mean pairwise differentiation was high (*F*_ST_ = 0.27 ± 0.00: Nei's *D*_ST_ = 0.326 ± 0.02) (Tables S2 and S3, Figure S4). South Coastal populations were particularly differentiated from others with an average *F*_ST_ from all other populations of 0.43 ± 0.001 (Figure S4, Table S2). Consistent with high *F*_ST_ values, AMOVA indicated that a high level (26.9%) of variation was maintained among populations. The mean number of effective migrants per generation (Nm) was 0.48 ± 0.001 across all populations, 0.443 ± 0.07 within North Coastal populations, 0.411 ± 0.09 within South Coastal populations, and 0.96 ± 0.05 within the Plateau cluster (Table S2).

There was a weak signal of isolation by distance across all 28 populations (*r* ^2^ = 0.037, *p* = .047). Within genetic groups, there was a pattern of increasing genetic differentiation with distance in the South Coastal cluster, but the relationship was not significant, likely a consequence of small sample size (*r* ^2^ = 0.81 *p* = .09). There was no evidence of isolation by distance within the North Coastal and Plateau clusters, respectively (North Coastal *r* ^2^ = 0.094 *p* = .06; Plateau *r* ^2^ = 0.0002 *p* = .50).

## 4 DISCUSSION

Investigation of historical and contemporary generic variation within the widespread species *B. sessilis* that spans the biogeographical boundary created by the Darling Fault shows genetic structure consistent with this geomorphological feature. Our analysis identified phylogeographical structure concordant with the uplift of the Darling Plateau and its separation from the SCP, demonstrating the influence of landscape processes on the evolutionary history of *B. sessilis*. Analysis of contemporary genetic relationships did not support our hypothesis of extensive gene flow across this widespread species. Three distinct genetic clusters were identified and populations within all three were at times highly differentiated and characterized by significant *F*_IS_ values that may indicate the occurrence of inbreeding.

### 4.1 The impact of palaeogeographical processes

Phylogeographic analysis of *Banksia sessilis* revealed two ancient phylogeographic lineages that diverged in the Pliocene (approx. 3.3 Ma). One lineage was restricted to lateritic soils of the Darling Plateau and Geraldton Sandplains and the other to non‐lateritic sands and limestones of the SCP and Southern Coastline. Interestingly, this structural pattern was not observed in the nuclear microsatellite data that showed three genetic clusters concordant with populations located on the Darling Plateau (lateritic), along the Northern Coastline (lateritic and non‐lateritic), and along the Southern Coastline (non‐lateritic). If the divergence of populations observed in the cpDNA were due to ecotype variation, where local adaptation (e.g., to the different substrate types) had resulted in incompatible lineages (Hufford & Mazer, 2003) then we would expect to see this pattern reflected in the nuclear microsatellite data. In contrast, we observed different structural genetic patterns in the contemporary nuclear data and observed gene flow between populations assigned to the different cpDNA lineages. This suggests that the geomorphological history of the Western Australian landscape during the Pliocene, in which the presence on laterite can be viewed as a proxy for landscape age and stability, is likely to be responsible for the observed cpDNA divergence, rather than other factors such as localized adaptation.

Laterite formation in Western Australia occurred throughout the Oligocene and Miocene (Johnstone et al., 1973; Mulcahy, 1960) when southern Australia was covered by closed rainforest (Bettenay, 1984). Its presence is indicative of extremely old and stable geological landscapes. The lateritic granites and duplex soils of the Darling Plateau have been relatively undisturbed by tectonism and were not impacted by sea level changes associated with the Quaternary glacial cycles (Bettenay, 1984). Parts of the Geraldton Sandplains are also ancient in origin and the populations Burma (BUR) and Waggrakine (WAG) in the northernmost locations sit atop lateritic mesas that represent the western edge of the dissected Victoria Plateau, an ancient mesa that underwent laterite development during the Oligocene (Department of Conservation and Land Management, 1998). The Geraldton Sandplains were prone to inundation during the Quaternary, but the raised profile of these lateritic features would have provided stability for plant populations during this time. The Lesueur (LES) population to the south of Burma and Waggrakine also sits atop a lateritic mesa that overlays residual sands (Playford et al., 1976) reaching an elevation of approx. 300 m. This altitude protected resident populations from rising sea levels that impacted flora on the younger coastal sands to the west. Thus, populations of the lateritic clade, whether found on the Darling Plateau or Geraldton Sandplains experienced similar biogeographic histories of landscape stability.

In contrast to the lateritic landscapes of the Darling Plateau and Geraldton Sandplains, coastal sands and limestones associated with the SCP and southern coastline are generally much younger in age and are therefore not laterized. These alluvial and eolian deposits range in age from the late Pliocene along the eastern margin of the SCP (McArthur & Bettenay, 1974) to recent dunes formed in the Holocene along the western margin of the SCP (McArthur & Bettenay, 1974) and along the southern Australian coastline (Short, 1988). This distinction between lateritic and non‐lateritic phylogeographic patterning is particularly obvious when looking at the Green head Rd (GHR) population. Green head Rd, although near to the lateritic Lesueur (LES) population, occurs on the low‐lying Illyarrie Vegetation system that overlies coastal limestone (Beard, 1979; Speck, 1958). Populations on these younger, low‐lying sediments would have been more heavily impacted by the marine inundations of the Pleistocene, impacting connectivity with populations occurring on lateritic sediments and strengthening patterns of divergence between the two clades.

The cpDNA data suggest both lineages of *B. sessilis* are old and relatively stable; neither show evidence of rapid demographic expansion that might be anticipated if there had been a sudden exploitation of a new environment made available by the uplifting of the Perth basin or due to a change in climatic conditions. Instead, tests of neutrality and mismatch analysis indicate historical stability and very low generational gene exchange in both clades (Fu, 1997; Ray et al., 2003; Tajima, 1989). Similarly, both the Bayesian and Parsimony analyses were inconsistent with sudden demographic expansion, with long‐branch lengths observed across both clades in the phylogenetic tree and a lack of a starlike phylogenetic pattern in the Parsimony network. Although no indications of sudden demographic expansions were observed, the data did suggest some spatial expansion had occurred within each clade. *Banksia sessilis* responds favorably to disturbance and aggressively colonizes new or disturbed habitats opened up both on historical timeframes through climate change and on a contemporary time scale through changing fire regimes and land use practices (Rockel et al., 1982).

The number of cpDNA haplotypes (*n* = 16) identified was high in comparison to other south‐western Australian *Banksia* species (*B. arborea*: *n* = 5 and *B. biterax*: *n* = 7) (Bradbury et al., 2019; Nistelberger et al., 2015). This is not unexpected given the widespread distribution of the species (Llorens et al., 2017; Loveless & Hamrick, 1984). The high levels of diversity and divergence evident in both cpDNA lineages suggest independent evolutionary histories of the two clades. We propose that physical isolation of *B. sessilis* populations, due to the uplift of the Darling Plateau combined with the impacts of subsequent sea level changes on populations present on the younger nonlaterized substrates, have driven patterns of deep genetic structure in this species.

Both the haplotype network and phylogenetic tree show the lateritic clade to further subdivide populations located on the Geraldton Sandplains from those that occur on the Darling Plateau, although statistical support for this was poor in the Bayesian phylogenetic analysis. Populations from the Geraldton Sandplains are also characterized by a high diversity of haplotypes given the limited geographic area from which these populations were sampled. This pattern of higher genetic diversity combined with the central positioning of these haplotypes in the Median Joining network suggest that the ancestral origins of *B. sessilis* may have been located within the Geraldton Sandplains. These data are also consistent with the nuclear microsatellite data that showed North Coastal populations had significantly higher levels of genetic diversity and align with the theory of sandplain heaths of the Geraldton Sandplains acting as a “biodiversity pump” to surrounding regions (Cardillo & Pratt, 2013; Hopper & Gioia, 2004).

There was one exception to the assignment of populations to clades based on substrate. The Jarrahdale (JAR) population occurs on laterite on the western edge of the Darling Scarp but is also close to non‐lateritic populations to the west. The Jarrahdale population possessed cpDNA haplotypes that were more closely related to non‐lateritic haplotypes on the SCP than to those on the lateritic Darling Plateau, suggesting the ancestor to this marginal population derived from the SCP. Subsequent gene flow with surrounding Darling Plateau populations would provide an explanation for the grouping of this population with other Plateau populations in the nuclear data.

Although we were not able to reliably assign populations to varietal status, we note that the separation between the lateritic clade and the non‐lateritic clade roughly corresponds to the substrate descriptions for the four varieties, two (var. *sessilis* and var. *flabellifolia*) largely on lateritic soils and two (var. *cordata* and var. *cygnorum*) on coastal and sands over limestone. This suggests that occurrence on substrate is a more reliable identifier of historical relationship than morphology.

### 4.2 Contemporary genetic connectivity

Species with widespread distributions are often expected to exhibit higher genetic diversity and less genetic structure than species with restricted distributions owing to increased opportunity for genetic connectivity among populations and the ability to maintain larger effective population sizes (Loveless & Hamrick, 1984). Despite the widespread distribution of *Banksia sessilis,* there was often evidence of limited gene flow, with moderate genetic structure observed among populations within the North Coastal and Plateau clusters and strong genetic structure within the South Coastal populations.

The observation of significant *F*_IS_ values, that can indicate inbreeding, in over half of the populations sampled also suggests pollen dispersal is often restricted. *Banksia sessilis* produces copious amounts of gravity dispersed seed that germinates the following wet season (Lamont et al., 1998). This is likely to result in sibship clusters within populations that can lead to inbreeding when birds forage in a nearest‐neighbor configuration as is often observed (Levin & Kerster, 1974; Webb, 1998). Indeed, relatedness estimates indicate that despite actively avoiding sampling neighboring plants from within populations, we have still captured related individuals, indicating wider neighborhood structure within populations. High inbreeding estimates can also arise when selfing is a feature of the mating system. Self‐compatibility is often limited among the banksias (Goldingay & Carthew, 1998) and has previously been suggested as rare in a *B. sessilis* population located on the Darling Scarp (Lamont et al., 1998). Yet selfing has been shown to vary within species (Coates et al., 2013; Llorens et al., 2012; Sampson et al., 1994; Vaughton, 1988) and evaluating the mating system operating in a range of populations throughout the distribution would be useful. *F*_IS_ estimates were particularly high among the more geographically isolated South Coastal and North Coastal populations, probably reflecting lowered rates of pollen immigration into those populations. The isolated South Coastal populations also showed evidence of genetic drift, with significantly lower levels of genetic diversity as well as strong genetic differentiation.

In addition to population isolation, the differences in genetic diversity, structure, and inbreeding observed among the genetic clusters may reflect variation in pollinator abundance and activity associated with different habitats, and/or plant density associated with fire regimes, among the North Coastal, South Coastal, and Plateau environments. We had no reason to expect influence of these factors in *B. sessilis,* and it would be interesting to evaluate the variability of these factors across the distribution and their influence on genetic patterns, particularly in populations in the North Coastal and South Coastal areas compared to those on the Plateau. The strong genetic structure among genetic clusters should be taken into consideration when utilizing this species in restoration, as it is contrary to that generally expected of a widespread species.

## CONFLICT OF INTEREST

None declared.

## AUTHOR CONTRIBUTIONS

**Heidi Maria Nistelberger:** Data curation (supporting); formal analysis (lead); investigation (lead); writing–original draft (lead). **Sarah‐Louise Tapper:** Data curation (equal); formal analysis (equal); investigation (equal). **David J. Coates:** Conceptualization (lead); writing–review and editing (supporting). **Shelley L. McArthur:** Data curation (equal); formal analysis (supporting). **Margaret Byrne:** Conceptualization (lead); funding acquisition (lead); project administration (equal); resources (equal); writing–review and editing (supporting).

## Supporting information

Supplementary Material

## Data Availability

DNA sequence data are available at Genbank KP331400‐KP33141; MK478025‐MK478041 (see Table S4). Microsatellite genotype data are available at Figshare https://doi.org/10.6084/m9.figshare.11905626.
