# Supplementary material for: As old as the hills: Pliocene palaeogeographical processes influence patterns of genetic structure in the widespread, common shrub Banksia sessilis
Source: Ecol Evol. 2020 Dec 28;11(2):1069–82. doi: 10.1002/ece3.7127 (PMC7820165; doi:10.1002/ece3.7127)
Supplement: Supplementary file 1 — Supplementary Material [file ECE3-11-1069-s001.DOC]

**SUPPLEMENTARY MATERIAL: As old as the hills: Pliocene palaeogeographical processes influence patterns of genetic structure in the widespread, common shrub *Banksia sessilis*.**

HM Nistelberger*, S Tapper, DJ Coates, SL McArthur, M Byrne

Table S1. Proportion of membership of each *Banksia sessilis* population to the designated number of clusters as tested in STRUCTURE: K=3 (main results), K=2 and K=4.

| *K =3* |  |  |  | K =2 |  |  | K =4 |  |  |  |  |
| --- | --- | --- | --- | --- | --- | --- | --- | --- | --- | --- | --- |
| *POP* | *COASTAL N* | *COASTAL S* | *PLATEAU* | POP | Cluster 1 | Cluster 2 | POP | Cluster 1 | Cluster 2 | Cluster 3 | Cluster 4 |
| *BAN* | *0.011* | *0.008* | *0.981* | BAN | 0.196 | 0.804 | BAN | 0.695 | 0.012 | 0.279 | 0.014 |
| *BUR* | *0.919* | *0.017* | *0.064* | BUR | 0.991 | 0.009 | BUR | 0.033 | 0.83 | 0.121 | 0.016 |
| *CRA* | *0.007* | *0.004* | *0.989* | CRA | 0.098 | 0.902 | CRA | 0.757 | 0.008 | 0.229 | 0.005 |
| *DEL* | *0.085* | *0.018* | *0.897* | DEL | 0.335 | 0.665 | DEL | 0.718 | 0.081 | 0.186 | 0.014 |
| *DON* | *0.021* | *0.015* | *0.963* | DON | 0.987 | 0.013 | DON | 0.065 | 0.009 | 0.915 | 0.01 |
| *GAP* | *0.021* | *0.973* | *0.007* | GAP | 0.009 | 0.991 | GAP | 0.006 | 0.019 | 0.008 | 0.966 |
| *GHR* | *0.919* | *0.022* | *0.059* | GHR | 0.987 | 0.013 | GHR | 0.045 | 0.835 | 0.097 | 0.023 |
| *GRA* | *0.012* | *0.981* | *0.008* | GRA | 0.98 | 0.02 | GRA | 0.008 | 0.012 | 0.01 | 0.969 |
| *HES* | *0.007* | *0.005* | *0.987* | HES | 0.927 | 0.073 | HES | 0.189 | 0.006 | 0.801 | 0.005 |
| *HOT* | *0.027* | *0.02* | *0.953* | HOT | 0.98 | 0.02 | HOT | 0.678 | 0.02 | 0.282 | 0.02 |
| *JAR* | *0.01* | *0.021* | *0.969* | JAR | 0.964 | 0.036 | JAR | 0.084 | 0.007 | 0.896 | 0.014 |
| *JULI* | *0.071* | *0.01* | *0.919* | JULI | 0.99 | 0.01 | JULI | 0.069 | 0.042 | 0.882 | 0.007 |
| *KOJ* | *0.006* | *0.006* | *0.988* | KOJ | 0.977 | 0.023 | KOJ | 0.836 | 0.006 | 0.147 | 0.012 |
| *LES* | *0.774* | *0.009* | *0.217* | LES | 0.02 | 0.98 | LES | 0.142 | 0.601 | 0.246 | 0.011 |
| *MIL* | *0.007* | *0.988* | *0.004* | MIL | 0.99 | 0.01 | MIL | 0.005 | 0.008 | 0.005 | 0.982 |
| *NY* | *0.942* | *0.021* | *0.037* | NY | 0.011 | 0.989 | NY | 0.022 | 0.906 | 0.049 | 0.024 |
| *OBR* | *0.027* | *0.006* | *0.967* | OBR | 0.989 | 0.011 | OBR | 0.479 | 0.019 | 0.495 | 0.007 |
| *POL* | *0.007* | *0.005* | *0.988* | POL | 0.009 | 0.991 | POL | 0.967 | 0.006 | 0.021 | 0.006 |
| *PRE* | *0.985* | *0.009* | *0.007* | PRE | 0.045 | 0.955 | PRE | 0.007 | 0.976 | 0.008 | 0.009 |
| *SAB* | *0.051* | *0.011* | *0.939* | SAB | 0.969 | 0.031 | SAB | 0.933 | 0.023 | 0.034 | 0.01 |
| *SAN* | *0.04* | *0.019* | *0.94* | SAN | 0.983 | 0.017 | SAN | 0.185 | 0.026 | 0.778 | 0.011 |
| *STR* | *0.007* | *0.016* | *0.978* | STR | 0.951 | 0.049 | STR | 0.808 | 0.007 | 0.169 | 0.016 |
| *SUG* | *0.004* | *0.991* | *0.005* | SUG | 0.991 | 0.009 | SUG | 0.006 | 0.004 | 0.004 | 0.986 |
| *UDU* | *0.057* | *0.026* | *0.917* | UDU | 0.083 | 0.917 | UDU | 0.083 | 0.026 | 0.871 | 0.021 |
| *UNI* | *0.004* | *0.005* | *0.99* | UNI | 0.132 | 0.868 | UNI | 0.927 | 0.005 | 0.061 | 0.007 |
| *WAG* | *0.913* | *0.02* | *0.066* | WAG | 0.948 | 0.052 | WAG | 0.048 | 0.866 | 0.064 | 0.021 |
| *WHI* | *0.982* | *0.006* | *0.012* | WHI | 0.668 | 0.332 | WHI | 0.011 | 0.965 | 0.017 | 0.006 |
| *WIL* | *0.013* | *0.019* | *0.967* | WIL | 0.934 | 0.066 | WIL | 0.582 | 0.014 | 0.387 | 0.017 |

Table S2. Pairwise *Banksia sessilis* population F_ST_ values based on nine nuclear microsatellite loci lower diagonal (all comparisons significant p <0.05). Pairwise estimates of the number of effective migrants (Nm) upper diagonal.

|  | BAN | BUR | CRA | DEL | DON | GAP | GHR | GRA | HES | HOT | JAR | JULI | KOJ | LES | MIL | NY | OBR | POL | PRE | SAB | SAN | STR | SUG | UDU | UNI | WAG | WHI | WIL |
| --- | --- | --- | --- | --- | --- | --- | --- | --- | --- | --- | --- | --- | --- | --- | --- | --- | --- | --- | --- | --- | --- | --- | --- | --- | --- | --- | --- | --- |
| BAN | 0.00 | 0.20 | 1.64 | 1.61 | 0.56 | 0.06 | 0.26 | 0.11 | 0.53 | 1.63 | 0.65 | 0.87 | 1.44 | 0.30 | 0.09 | 0.20 | 0.88 | 0.87 | 0.15 | 0.42 | 1.02 | 1.52 | 0.08 | 0.68 | 0.77 | 0.22 | 0.20 | 1.48 |
| BUR | 0.25 | 0.00 | 0.21 | 0.49 | 0.20 | 0.08 | 0.61 | 0.14 | 0.21 | 0.24 | 0.21 | 0.34 | 0.18 | 0.26 | 0.14 | 0.47 | 0.23 | 0.15 | 0.26 | 0.14 | 0.28 | 0.18 | 0.11 | 0.32 | 0.17 | 0.78 | 0.22 | 0.23 |
| CRA | 0.10 | 0.23 | 0.00 | 1.82 | 0.65 | 0.07 | 0.25 | 0.12 | 1.11 | 2.36 | 0.81 | 0.72 | 2.82 | 0.29 | 0.09 | 0.21 | 1.44 | 0.85 | 0.14 | 0.68 | 1.19 | 1.87 | 0.09 | 0.80 | 1.45 | 0.23 | 0.18 | 1.07 |
| DEL | 0.06 | 0.13 | 0.06 | 0.00 | 0.67 | 0.07 | 0.42 | 0.12 | 0.87 | 1.60 | 0.89 | 0.87 | 1.73 | 0.37 | 0.11 | 0.26 | 1.62 | 0.67 | 0.21 | 0.57 | 1.21 | 0.67 | 0.10 | 1.27 | 0.89 | 0.31 | 0.29 | 1.41 |
| DON | 0.23 | 0.26 | 0.21 | 0.16 | 0.00 | 0.08 | 0.28 | 0.16 | 0.33 | 0.53 | 0.26 | 0.51 | 0.47 | 0.44 | 0.12 | 0.18 | 0.50 | 0.27 | 0.17 | 0.31 | 0.29 | 0.63 | 0.11 | 0.83 | 0.40 | 0.19 | 0.17 | 0.27 |
| GAP | 0.55 | 0.43 | 0.52 | 0.46 | 0.54 | 0.00 | 0.12 | 0.21 | 0.07 | 0.07 | 0.07 | 0.08 | 0.07 | 0.08 | 0.29 | 0.13 | 0.07 | 0.07 | 0.09 | 0.06 | 0.08 | 0.07 | 0.17 | 0.09 | 0.07 | 0.11 | 0.08 | 0.07 |
| GHR | 0.23 | 0.07 | 0.22 | 0.14 | 0.25 | 0.43 | 0.00 | 0.19 | 0.21 | 0.30 | 0.26 | 0.41 | 0.18 | 0.41 | 0.15 | 1.60 | 0.30 | 0.18 | 0.31 | 0.14 | 0.33 | 0.23 | 0.12 | 0.43 | 0.24 | 0.58 | 0.40 | 0.22 |
| GRA | 0.43 | 0.31 | 0.40 | 0.35 | 0.42 | 0.41 | 0.27 | 0.00 | 0.10 | 0.11 | 0.11 | 0.14 | 0.11 | 0.11 | 0.53 | 0.15 | 0.12 | 0.10 | 0.12 | 0.08 | 0.12 | 0.15 | 0.85 | 0.16 | 0.13 | 0.12 | 0.11 | 0.10 |
| HES | 0.30 | 0.29 | 0.20 | 0.18 | 0.39 | 0.61 | 0.31 | 0.50 | 0.00 | 1.00 | 1.66 | 0.59 | 0.89 | 0.18 | 0.09 | 0.18 | 0.62 | 0.47 | 0.17 | 0.46 | 1.16 | 0.42 | 0.08 | 0.61 | 0.57 | 0.19 | 0.20 | 0.61 |
| HOT | 0.14 | 0.20 | 0.10 | 0.06 | 0.25 | 0.53 | 0.18 | 0.41 | 0.20 | 0.00 | 2.12 | 1.14 | 2.57 | 0.36 | 0.10 | 0.20 | 1.82 | 0.87 | 0.15 | 0.92 | 1.60 | 1.06 | 0.10 | 0.98 | 1.08 | 0.26 | 0.20 | 2.08 |
| JAR | 0.22 | 0.23 | 0.19 | 0.12 | 0.34 | 0.57 | 0.20 | 0.44 | 0.15 | 0.08 | 0.00 | 0.89 | 0.69 | 0.23 | 0.09 | 0.18 | 1.16 | 0.56 | 0.12 | 0.46 | 1.21 | 0.43 | 0.09 | 0.83 | 0.72 | 0.20 | 0.18 | 0.97 |
| JULI | 0.15 | 0.16 | 0.12 | 0.08 | 0.22 | 0.51 | 0.14 | 0.38 | 0.20 | 0.08 | 0.11 | 0.00 | 0.60 | 0.35 | 0.11 | 0.28 | 1.04 | 0.52 | 0.15 | 0.28 | 1.66 | 0.52 | 0.11 | 2.14 | 0.59 | 0.28 | 0.24 | 1.47 |
| KOJ | 0.14 | 0.27 | 0.06 | 0.08 | 0.26 | 0.52 | 0.28 | 0.43 | 0.21 | 0.10 | 0.22 | 0.16 | 0.00 | 0.21 | 0.10 | 0.15 | 1.34 | 0.67 | 0.12 | 1.28 | 1.55 | 1.61 | 0.09 | 0.74 | 1.95 | 0.22 | 0.18 | 2.13 |
| LES | 0.23 | 0.20 | 0.24 | 0.15 | 0.20 | 0.49 | 0.19 | 0.41 | 0.36 | 0.20 | 0.28 | 0.21 | 0.28 | 0.00 | 0.09 | 0.23 | 0.33 | 0.18 | 0.18 | 0.22 | 0.22 | 0.21 | 0.09 | 0.35 | 0.19 | 0.41 | 0.19 | 0.23 |
| MIL | 0.43 | 0.29 | 0.40 | 0.34 | 0.42 | 0.36 | 0.31 | 0.24 | 0.51 | 0.42 | 0.47 | 0.39 | 0.41 | 0.41 | 0.00 | 0.15 | 0.10 | 0.08 | 0.10 | 0.07 | 0.11 | 0.11 | 0.42 | 0.12 | 0.09 | 0.11 | 0.10 | 0.10 |
| NY | 0.26 | 0.09 | 0.25 | 0.17 | 0.28 | 0.41 | 0.03 | 0.32 | 0.34 | 0.22 | 0.26 | 0.19 | 0.30 | 0.22 | 0.31 | 0.00 | 0.22 | 0.17 | 0.32 | 0.11 | 0.25 | 0.15 | 0.11 | 0.28 | 0.17 | 0.49 | 0.35 | 0.19 |
| OBR | 0.14 | 0.22 | 0.08 | 0.05 | 0.19 | 0.53 | 0.19 | 0.40 | 0.23 | 0.09 | 0.14 | 0.09 | 0.10 | 0.20 | 0.41 | 0.24 | 0.00 | 0.62 | 0.12 | 0.46 | 0.96 | 0.93 | 0.10 | 1.20 | 1.95 | 0.26 | 0.18 | 1.20 |
| POL | 0.18 | 0.32 | 0.16 | 0.16 | 0.33 | 0.54 | 0.29 | 0.44 | 0.34 | 0.18 | 0.28 | 0.23 | 0.18 | 0.30 | 0.46 | 0.30 | 0.20 | 0.00 | 0.13 | 0.49 | 0.47 | 0.56 | 0.10 | 0.51 | 0.60 | 0.17 | 0.12 | 0.95 |
| PRE | 0.34 | 0.22 | 0.36 | 0.25 | 0.35 | 0.46 | 0.22 | 0.38 | 0.40 | 0.34 | 0.39 | 0.31 | 0.39 | 0.27 | 0.40 | 0.22 | 0.37 | 0.37 | 0.00 | 0.11 | 0.17 | 0.09 | 0.10 | 0.19 | 0.10 | 0.21 | 0.78 | 0.12 |
| SAB | 0.24 | 0.29 | 0.17 | 0.16 | 0.30 | 0.53 | 0.30 | 0.44 | 0.30 | 0.16 | 0.27 | 0.26 | 0.14 | 0.26 | 0.45 | 0.32 | 0.22 | 0.21 | 0.36 | 0.00 | 0.49 | 0.40 | 0.07 | 0.41 | 0.35 | 0.15 | 0.13 | 0.53 |
| SAN | 0.16 | 0.21 | 0.11 | 0.08 | 0.31 | 0.53 | 0.20 | 0.42 | 0.16 | 0.08 | 0.13 | 0.08 | 0.11 | 0.27 | 0.42 | 0.24 | 0.14 | 0.25 | 0.33 | 0.21 | 0.00 | 0.61 | 0.10 | 1.14 | 0.91 | 0.30 | 0.28 | 1.34 |
| STR | 0.17 | 0.29 | 0.08 | 0.17 | 0.26 | 0.56 | 0.28 | 0.40 | 0.33 | 0.19 | 0.29 | 0.22 | 0.11 | 0.32 | 0.43 | 0.32 | 0.15 | 0.22 | 0.44 | 0.24 | 0.22 | 0.00 | 0.12 | 0.44 | 2.51 | 0.19 | 0.12 | 0.66 |
| SUG | 0.49 | 0.38 | 0.45 | 0.40 | 0.49 | 0.41 | 0.37 | 0.24 | 0.57 | 0.46 | 0.51 | 0.45 | 0.47 | 0.44 | 0.25 | 0.37 | 0.45 | 0.46 | 0.45 | 0.49 | 0.48 | 0.47 | 0.00 | 0.12 | 0.12 | 0.09 | 0.08 | 0.10 |
| UDU | 0.14 | 0.16 | 0.14 | 0.07 | 0.15 | 0.47 | 0.13 | 0.33 | 0.20 | 0.10 | 0.11 | 0.06 | 0.16 | 0.18 | 0.36 | 0.18 | 0.09 | 0.18 | 0.25 | 0.20 | 0.13 | 0.21 | 0.40 | 0.00 | 0.68 | 0.36 | 0.28 | 0.96 |
| UNI | 0.19 | 0.27 | 0.11 | 0.12 | 0.28 | 0.56 | 0.24 | 0.41 | 0.31 | 0.15 | 0.22 | 0.17 | 0.12 | 0.30 | 0.44 | 0.29 | 0.08 | 0.22 | 0.44 | 0.28 | 0.20 | 0.08 | 0.47 | 0.16 | 0.00 | 0.19 | 0.14 | 0.73 |
| WAG | 0.22 | 0.07 | 0.20 | 0.14 | 0.24 | 0.41 | 0.09 | 0.33 | 0.30 | 0.17 | 0.23 | 0.16 | 0.23 | 0.14 | 0.32 | 0.12 | 0.18 | 0.28 | 0.25 | 0.26 | 0.20 | 0.27 | 0.38 | 0.14 | 0.25 | 0.00 | 0.24 | 0.25 |
| WHI | 0.31 | 0.22 | 0.33 | 0.23 | 0.36 | 0.46 | 0.18 | 0.39 | 0.35 | 0.30 | 0.32 | 0.26 | 0.35 | 0.28 | 0.41 | 0.18 | 0.32 | 0.38 | 0.12 | 0.36 | 0.26 | 0.42 | 0.45 | 0.22 | 0.40 | 0.22 | 0.00 | 0.17 |
| WIL | 0.09 | 0.19 | 0.07 | 0.04 | 0.26 | 0.49 | 0.19 | 0.40 | 0.17 | 0.04 | 0.11 | 0.06 | 0.05 | 0.22 | 0.38 | 0.22 | 0.09 | 0.14 | 0.32 | 0.16 | 0.07 | 0.16 | 0.42 | 0.08 | 0.14 | 0.17 | 0.27 | 0.00 |

Table S3. Pairwise population values of Nei’s unbiased genetic distance (Nei, 1972) based on nine nuclear microsatellite markers in *Banksia sessilis*.

|  | BAN | BUR | CRA | DEL | DON | GAP | GHR | GRA | HES | HOT | JAR | JULI | KOJ | LES | MIL | NY | OBR | POL | PRE | SAB | SAN | STR | SUG | UDU | UNI | WAG | WHI | WIL |
| --- | --- | --- | --- | --- | --- | --- | --- | --- | --- | --- | --- | --- | --- | --- | --- | --- | --- | --- | --- | --- | --- | --- | --- | --- | --- | --- | --- | --- |
| BAN | 0.000 |  |  |  |  |  |  |  |  |  |  |  |  |  |  |  |  |  |  |  |  |  |  |  |  |  |  |  |
| BUR | 0.340 | 0.000 |  |  |  |  |  |  |  |  |  |  |  |  |  |  |  |  |  |  |  |  |  |  |  |  |  |  |
| CRA | 0.077 | 0.309 | 0.000 |  |  |  |  |  |  |  |  |  |  |  |  |  |  |  |  |  |  |  |  |  |  |  |  |  |
| DEL | 0.056 | 0.185 | 0.053 | 0.000 |  |  |  |  |  |  |  |  |  |  |  |  |  |  |  |  |  |  |  |  |  |  |  |  |
| DON | 0.195 | 0.304 | 0.165 | 0.136 | 0.000 |  |  |  |  |  |  |  |  |  |  |  |  |  |  |  |  |  |  |  |  |  |  |  |
| GAP | 0.952 | 0.700 | 0.809 | 0.753 | 0.744 | 0.000 |  |  |  |  |  |  |  |  |  |  |  |  |  |  |  |  |  |  |  |  |  |  |
| GHR | 0.289 | 0.088 | 0.272 | 0.177 | 0.260 | 0.600 | 0.000 |  |  |  |  |  |  |  |  |  |  |  |  |  |  |  |  |  |  |  |  |  |
| GRA | 0.620 | 0.415 | 0.516 | 0.488 | 0.467 | 0.377 | 0.318 | 0.000 |  |  |  |  |  |  |  |  |  |  |  |  |  |  |  |  |  |  |  |  |
| HES | 0.240 | 0.301 | 0.141 | 0.140 | 0.320 | 0.928 | 0.317 | 0.601 | 0.000 |  |  |  |  |  |  |  |  |  |  |  |  |  |  |  |  |  |  |  |
| HOT | 0.111 | 0.231 | 0.073 | 0.050 | 0.208 | 0.774 | 0.187 | 0.500 | 0.127 | 0.000 |  |  |  |  |  |  |  |  |  |  |  |  |  |  |  |  |  |  |
| JAR | 0.175 | 0.246 | 0.143 | 0.094 | 0.284 | 0.854 | 0.190 | 0.504 | 0.081 | 0.049 | 0.000 |  |  |  |  |  |  |  |  |  |  |  |  |  |  |  |  |  |
| JULI | 0.128 | 0.184 | 0.102 | 0.066 | 0.177 | 0.750 | 0.153 | 0.480 | 0.131 | 0.056 | 0.075 | 0.000 |  |  |  |  |  |  |  |  |  |  |  |  |  |  |  |  |
| KOJ | 0.111 | 0.358 | 0.044 | 0.071 | 0.218 | 0.769 | 0.356 | 0.559 | 0.144 | 0.071 | 0.165 | 0.130 | 0.000 |  |  |  |  |  |  |  |  |  |  |  |  |  |  |  |
| LES | 0.245 | 0.282 | 0.258 | 0.167 | 0.161 | 0.764 | 0.241 | 0.606 | 0.374 | 0.185 | 0.271 | 0.205 | 0.324 | 0.000 |  |  |  |  |  |  |  |  |  |  |  |  |  |  |
| MIL | 0.582 | 0.384 | 0.521 | 0.455 | 0.464 | 0.302 | 0.377 | 0.185 | 0.634 | 0.525 | 0.600 | 0.492 | 0.509 | 0.627 | 0.000 |  |  |  |  |  |  |  |  |  |  |  |  |  |
| NY | 0.329 | 0.123 | 0.319 | 0.224 | 0.321 | 0.537 | 0.032 | 0.397 | 0.380 | 0.244 | 0.272 | 0.215 | 0.406 | 0.295 | 0.369 | 0.000 |  |  |  |  |  |  |  |  |  |  |  |  |
| OBR | 0.121 | 0.274 | 0.062 | 0.049 | 0.143 | 0.839 | 0.221 | 0.502 | 0.168 | 0.066 | 0.094 | 0.072 | 0.076 | 0.193 | 0.543 | 0.288 | 0.000 |  |  |  |  |  |  |  |  |  |  |  |
| POL | 0.159 | 0.479 | 0.136 | 0.156 | 0.312 | 0.829 | 0.382 | 0.606 | 0.288 | 0.149 | 0.247 | 0.212 | 0.150 | 0.358 | 0.676 | 0.400 | 0.185 | 0.000 |  |  |  |  |  |  |  |  |  |  |
| PRE | 0.471 | 0.312 | 0.560 | 0.351 | 0.416 | 0.654 | 0.281 | 0.532 | 0.475 | 0.453 | 0.499 | 0.407 | 0.607 | 0.364 | 0.558 | 0.287 | 0.575 | 0.525 | 0.000 |  |  |  |  |  |  |  |  |  |
| SAB | 0.255 | 0.471 | 0.162 | 0.182 | 0.303 | 0.898 | 0.453 | 0.682 | 0.254 | 0.140 | 0.245 | 0.284 | 0.116 | 0.332 | 0.695 | 0.496 | 0.215 | 0.195 | 0.588 | 0.000 |  |  |  |  |  |  |  |  |
| SAN | 0.129 | 0.240 | 0.082 | 0.064 | 0.280 | 0.784 | 0.215 | 0.527 | 0.092 | 0.055 | 0.082 | 0.056 | 0.082 | 0.290 | 0.525 | 0.278 | 0.111 | 0.231 | 0.414 | 0.194 | 0.000 |  |  |  |  |  |  |  |
| STR | 0.134 | 0.361 | 0.054 | 0.145 | 0.191 | 0.813 | 0.313 | 0.440 | 0.244 | 0.139 | 0.225 | 0.181 | 0.074 | 0.349 | 0.488 | 0.393 | 0.107 | 0.169 | 0.726 | 0.203 | 0.173 | 0.000 |  |  |  |  |  |  |
| SUG | 0.721 | 0.554 | 0.608 | 0.555 | 0.601 | 0.356 | 0.464 | 0.171 | 0.784 | 0.577 | 0.650 | 0.573 | 0.624 | 0.630 | 0.174 | 0.476 | 0.589 | 0.589 | 0.657 | 0.770 | 0.652 | 0.536 | 0.000 |  |  |  |  |  |
| UDU | 0.138 | 0.227 | 0.132 | 0.074 | 0.120 | 0.769 | 0.168 | 0.454 | 0.155 | 0.088 | 0.076 | 0.047 | 0.149 | 0.205 | 0.515 | 0.245 | 0.076 | 0.182 | 0.354 | 0.238 | 0.119 | 0.195 | 0.573 | 0.000 |  |  |  |  |
| UNI | 0.141 | 0.309 | 0.070 | 0.093 | 0.203 | 0.773 | 0.237 | 0.424 | 0.214 | 0.098 | 0.143 | 0.122 | 0.072 | 0.297 | 0.501 | 0.309 | 0.046 | 0.170 | 0.664 | 0.248 | 0.144 | 0.044 | 0.506 | 0.125 | 0.000 |  |  |  |
| WAG | 0.289 | 0.096 | 0.254 | 0.193 | 0.258 | 0.663 | 0.112 | 0.494 | 0.326 | 0.195 | 0.250 | 0.176 | 0.294 | 0.170 | 0.457 | 0.155 | 0.215 | 0.402 | 0.382 | 0.402 | 0.226 | 0.329 | 0.603 | 0.187 | 0.264 | 0.000 |  |  |
| WHI | 0.421 | 0.320 | 0.489 | 0.317 | 0.477 | 0.695 | 0.214 | 0.592 | 0.391 | 0.387 | 0.364 | 0.311 | 0.527 | 0.412 | 0.643 | 0.222 | 0.464 | 0.618 | 0.121 | 0.625 | 0.300 | 0.677 | 0.719 | 0.303 | 0.566 | 0.353 | 0.000 |  |
| WIL | 0.079 | 0.270 | 0.065 | 0.045 | 0.253 | 0.798 | 0.257 | 0.587 | 0.119 | 0.033 | 0.083 | 0.048 | 0.042 | 0.268 | 0.523 | 0.297 | 0.080 | 0.124 | 0.488 | 0.160 | 0.049 | 0.133 | 0.589 | 0.083 | 0.103 | 0.236 | 0.392 | 0.000 |

Table S4. Genbank accession numbers for *Banksia sessilis* cpDNA sequence haplotypes

| **Sample** | **cpDNA clade** | **petB** |
| --- | --- | --- |
| *Banksia echinata* | na | MK478042 |
| *Banksia hewardiana* | na | MK478043 |
| ban1 | lateritic | KP331409.1 |
| bur1 | lateritic | KP331410.1 |
| bur8 | lateritic | KP331411.1 |
| ghr1 | non-lateritic | KP331412.1 |
| pre1 | non-lateritic | KP331413.1 |
| **Sample** |  | **trnS_trnG** |
| *Banksia echinata* | na | MK478044 |
| *Banksia hewardiana* | na | MK478045 |
| ban1 | lateritic | KP331400.1 |
| bur1 | lateritic | KP331401.1 |
| bur8 | lateritic | KP331402.1 |
| don1 | lateritic | KP331403.1 |
| gap1 | non-lateritic | KP331404.1 |
| ghr1 | non-lateritic | KP331405.1 |
| les1 | lateritic | KP331406.1 |
| ny1 | non-lateritic | KP331407.1 |
| pre1 | non-lateritic | KP331408.1 |
| **Sample** |  | **trnT-psbD** |
| *Banksia echinata* | na | MK478025 |
| *Banksia hewardiana* | na | MK478026 |
| ban1 | lateritic | MK478027 |
| ban24 | lateritic | MK478028 |
| bur1 | lateritic | MK478029 |
| bur17 | lateritic | MK478030 |
| cra1 | lateritic | MK478031 |
| del1 | lateritic | MK478032 |
| don1 | lateritic | MK478033 |
| gap1 | non-lateritic | MK478034 |
| ghr1 | non-lateritic | MK478035 |
| jar1 | non-lateritic | MK478036 |
| jar8 | non-lateritic | MK478037 |
| les1 | lateritic | MK478038 |
| ny1 | non-lateritic | MK478039 |
| pre1 | non-lateritic | MK478040 |
| wag17 | lateritic | MK478041 |


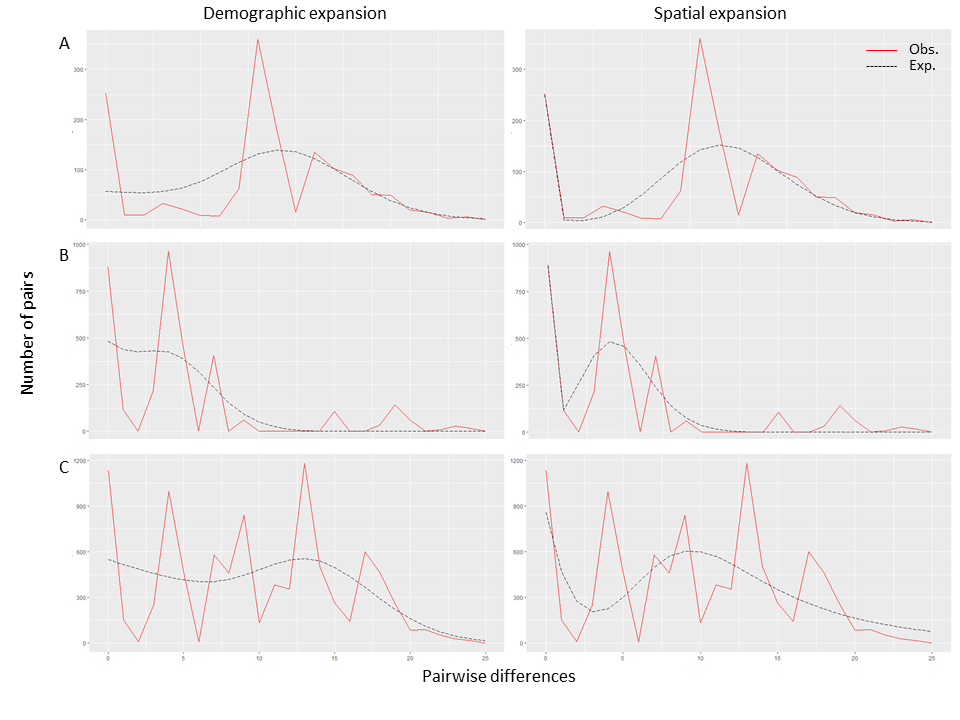


Fig. S1) Mismatch distributions under scenarios of demographic and spatial expansion for the A) non-lateritic clade, B) lateritic clade and C) all populations of *Banksia sessilis*.

A)


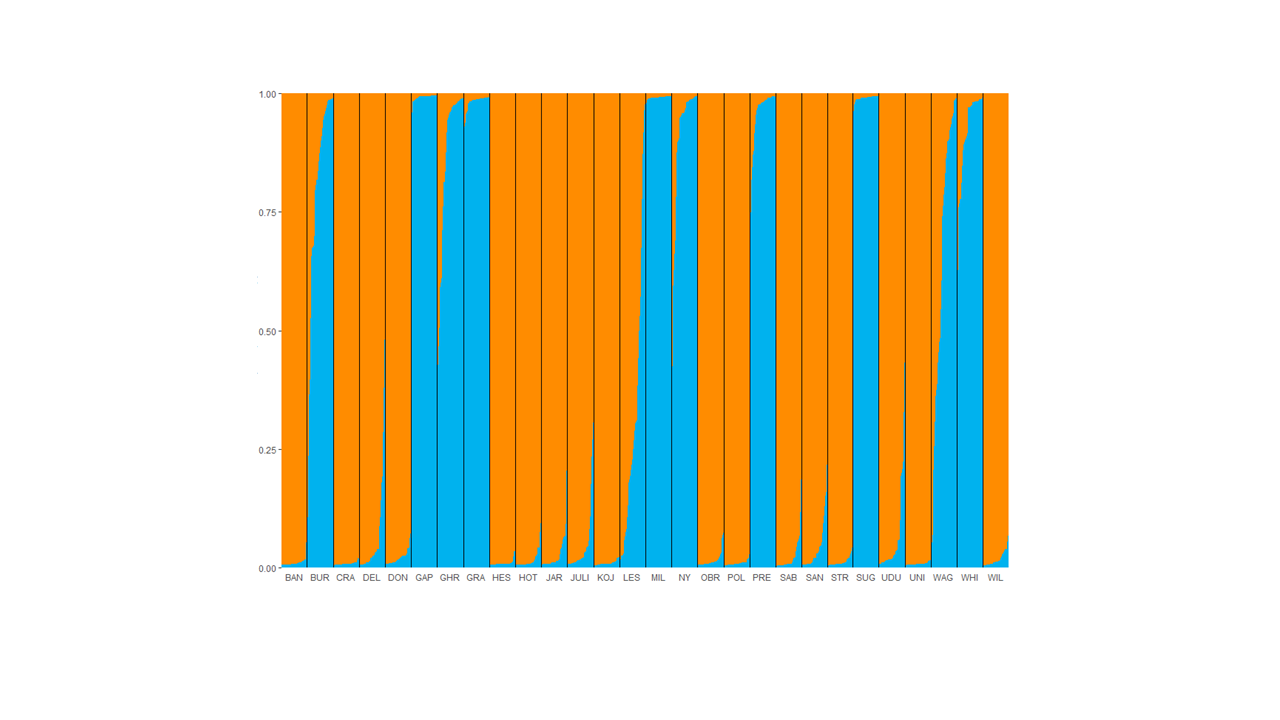


B)


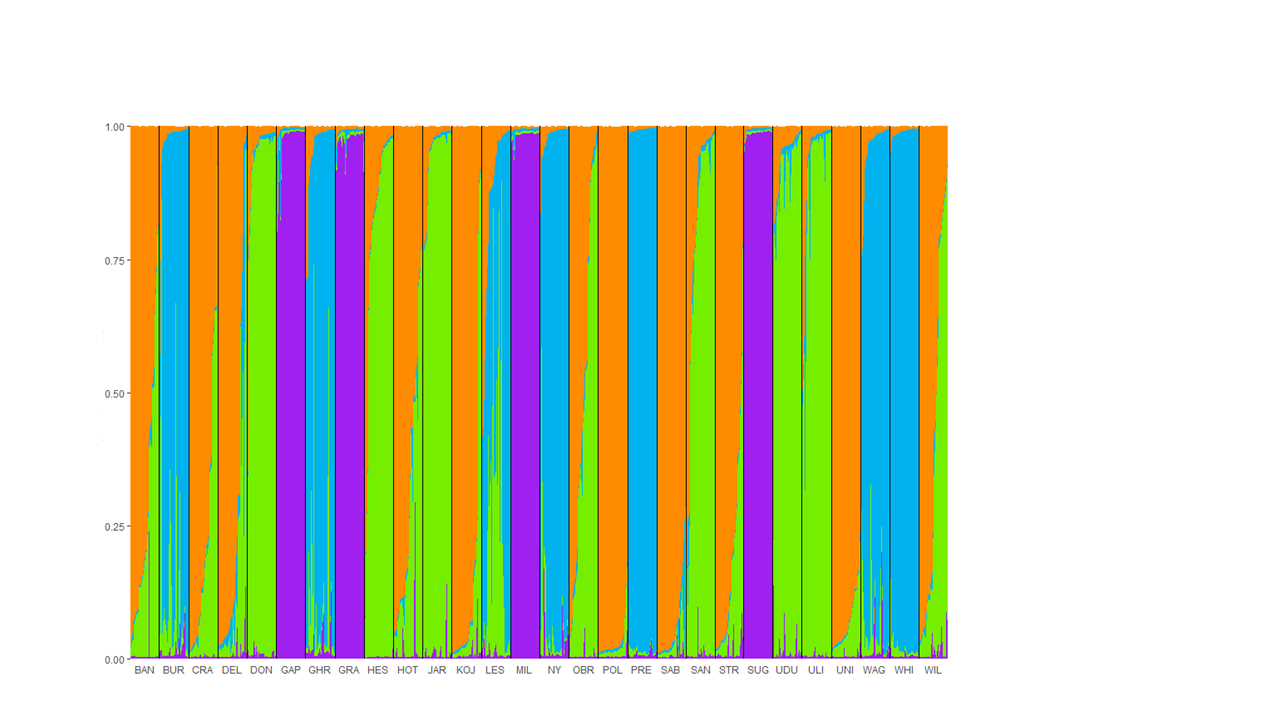


Fig. S2) Structure barplots showing the proportion of assignment of each individual to A) K = 2 and B) K = 4 genetic clusters.


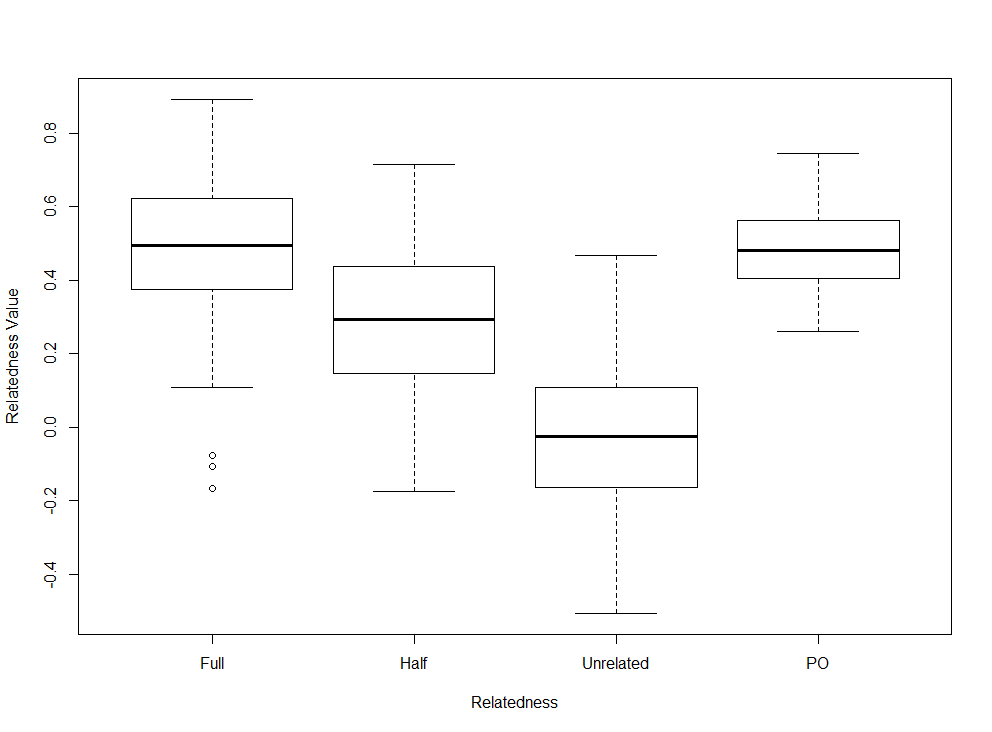


Fig. S3) Plot showing the relatedness values and confidence intervals of 100 simulated individuals of “full sibling”, “half sibling”, “unrelated” and “parent-offspring” relationship using the Wang (2002) estimator in the R package related.


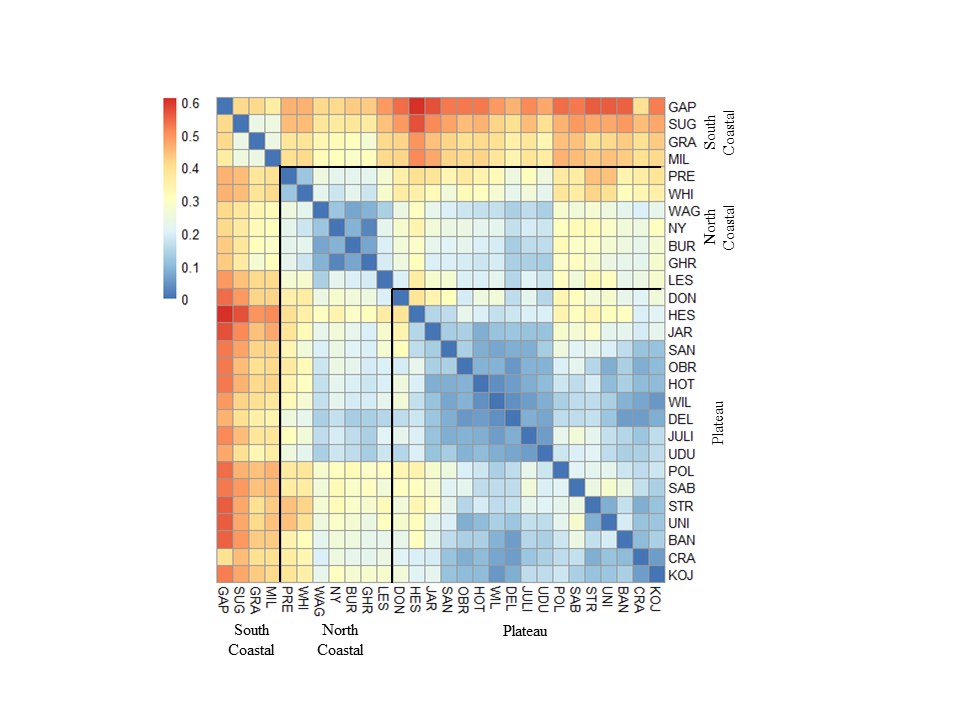


Fig. S4) Heatmap of population pairwise F_ST_ values based on nine microsatellite loci for populations of *Banksia sessilis*. Warmer colours represent higher genetic differentiation.
